# Mechanisms Underlying the Antidiabetic Activities of Polyphenolic Compounds: A Review

**DOI:** 10.3389/fphar.2021.798329

**Published:** 2021-12-14

**Authors:** Tina Nie, Garth J. S. Cooper

**Affiliations:** ^1^ School of Biological Sciences, Faculty of Science, the University of Auckland, Auckland, New Zealand; ^2^ The Maurice Wilkins Centre for Molecular Biodiscovery, Faculty of Science, the University of Auckland, Auckland, New Zealand; ^3^ Centre for Advanced Discovery and Experimental Therapeutics, Division of Cardiovascular Sciences, Faculty of Biology Medicine & Health, School of Medical Sciences, The University of Manchester, Manchester, United Kingdom

**Keywords:** amylin, IAPP, insulin, islet β-cells, flavonoids, polyphenol, natural products, type 2 diabetes

## Abstract

Polyphenolic compounds are thought to show considerable promise for the treatment of various metabolic disorders, including type 2 diabetes mellitus (T2DM). This review addresses evidence from *in vitro*, *in vivo*, and clinical studies for the antidiabetic effects of certain polyphenolic compounds. We focus on the role of cytotoxic human amylin (hA) aggregates in the pathogenesis of T2DM, and how polyphenols can ameliorate this process by suppressing or modifying their formation. Small, soluble amylin oligomers elicit cytotoxicity in pancreatic islet β-cells and may thus cause β-cell disruption in T2DM. Amylin oligomers may also contribute to oxidative stress and inflammation that lead to the triggering of β-cell apoptosis. Polyphenols may exert antidiabetic effects via their ability to inhibit hA aggregation, and to modulate oxidative stress, inflammation, and other pathways that are β-cell-protective or insulin-sensitizing. There is evidence that their ability to inhibit and destabilize self-assembly by hA requires aromatic molecular structures that bind to misfolding monomers or oligomers, coupled with adjacent hydroxyl groups present on single phenyl rings. Thus, these multifunctional compounds have the potential to be effective against the pleiotropic mechanisms of T2DM. However, substantial further research will be required before it can be determined whether a polyphenol-based molecular entity can be used as a therapeutic for type 2 diabetes.

## Introduction

Type 2 diabetes mellitus (T2DM) is a disorder of blood glucose regulation, characterized by hyperglycemia, insulin resistance, pancreatic amyloid deposition, and β-cell dysfunction (Ashcroft and Rorsman, 2012). T2DM has a significant impact on quality of life and is among the leading causes of mortality for adults worldwide (Saeedi et al., 2020). The impact of T2DM is amplified by the lack of currently available disease-modifying therapies for treatment of T2DM. Although important advances have been made in T2DM therapy, currently available drugs are only able to treat the symptoms of the disease and not the underlying etiopathogenic mechanisms (Padhi et al., 2020).

Under normal circumstances, the pancreas compensates for decreased insulin sensitivity by increasing β-cell mass and insulin secretion. However, in T2DM insulin secretion eventually fails to meet metabolic requirements. The underlying cause for this process is unknown, but has been postulated to relate to amyloid deposition, oxidative stress, or exposure to elevated free fatty acids (FFA), glucose or proinflammatory cytokines.

The hormone amylin (human amylin or hA; also known as islet amyloid polypeptide or IAPP) is said to be implicated in the pathogenesis of T2DM. Supporting evidence includes the presence of amyloid deposits composed primarily of hA being found in the pancreatic islets of most patients (>90%) with T2DM (Clark et al., 1987; Cooper et al., 1987). Oligomers of human amylin have been shown to elicit cytotoxicity and may contribute to the β-cell dysfunction seen in T2DM (Zhang et al., 2003).

Several inhibitors of *in vitro* amylin aggregation have been identified. These can be categorized into four major classes: naturally occurring small polyphenolic compounds, peptides, antibodies, and nanomaterials (Ladiwala et al., 2012; Tang et al., 2020). However, the exact mechanism of action of many inhibitors remains unclear, partly due to the difficulty of observing the early, small oligomer stage of the amylin aggregation process (Abedini et al., 2016; Samdin et al., 2021).

Polyphenolic compounds have received considerable attention for their potential to modulate numerous diseases, for example T2DM. A wide variety of polyphenols are abundant in various plant-based food sources and include flavonoids (which are further divided into several sub-classes), phenolic acids, and non-flavonoids (Sequeira & Poppitt, 2017). Accumulating evidence suggests that the molecular scaffold of polyphenols implicated in the suppression of cytotoxicity (including multiple phenol rings and hydroxyl functional groups) enables their wide range of activities. These include their ability to inhibit the aggregation of amyloidogenic peptides, oxidative stress, inflammation, and modulate various signaling pathways. This review will focus on the antidiabetic effects of natural polyphenolic compounds, their proposed mechanisms of actions and make suggestions for further research.

## The Role of Amylin in the Pathogenesis of β-cell Dysfunction and T2DM

Many factors, including elevated lipid levels, oxidative stress, and adipose-derived cytokines, are linked to insulin resistance (Wellen and Hotamisligil, 2005). However, β-cells can initially compensate for this by increasing insulin release, and it is a subsequent decline in β-cell function that leads to hyperglycemia and overt T2DM. The cause of β-cell dysfunction in T2DM remains uncertain; one key postulated mechanism is that it is due to aggregation of hA, making it an attractive potential therapeutic target.

Amylin is a β-cell hormone involved in the slowing of gastric emptying (Young, 2005), inhibition of insulin secretion (Silvestre et al., 1990), antagonizing insulin action in skeletal muscle (Leighton and Cooper, 1988), and suppression of appetite via modulation of neuropeptide signaling (Nie et al., 2019).

The hA molecule is a 37 amino acid peptide containing a disulfide bridge between Cys2 and Cys7 and an amidated C-terminal (Cooper et al., 1989). In its native physiological state, hA adopts a random coil conformation; however, in certain conditions hA can misfold and form oligomers that elongate and in turn form fibrils (Westermark et al., 1990). Due to small changes in the amino acid sequence between residues 20 and 29 (termed the amyloidogenic region), hA has this self-assembling property, whereas rat amylin does not (Cooper et al., 1989). Pancreatic amyloid has structural similarities to other amyloids including those formed by amyloid β in Alzheimer’s disease and α-synuclein in Parkinson’s disease, which are rich in β-sheets (Porat et al., 2006). Notwithstanding small changes, the peptides involved do not share substantive sequence identity, but contain aromatic residues that play roles in stabilizing these β-sheet structures (Gazit, 2002), possibly through π-π stacking of aromatic side chains between peptides in the C-terminal region (Wei et al., 2011).

**FIGURE 1:**
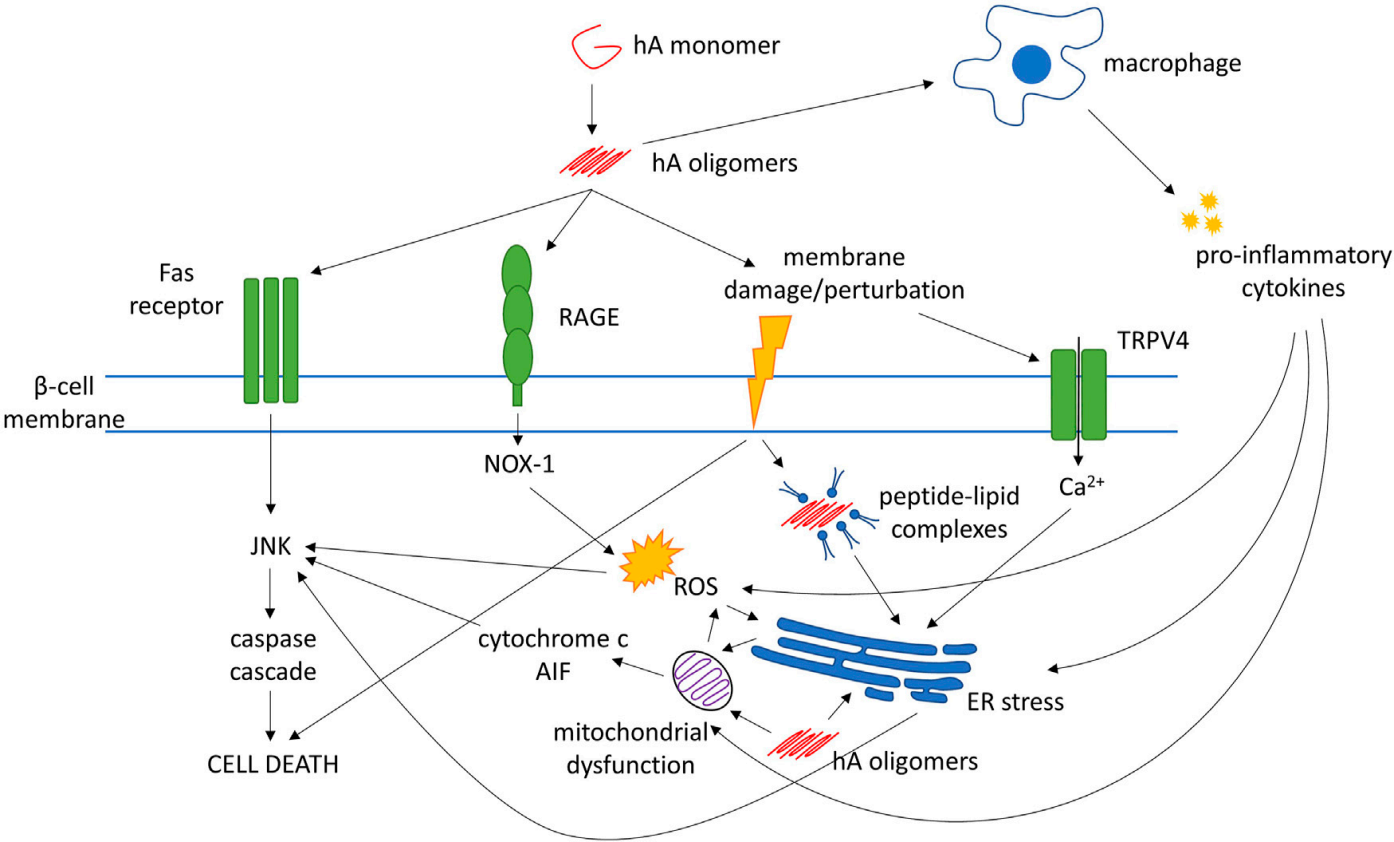
Mechanisms of human amylin (hA)-evoked β-cell death. Abbreviations: AIF, apoptosis-inducing factor; ER, endoplasmic reticulum; JNK, c-Jun N-terminal kinase; NOX-1, NADPH oxidase-1; RAGE, receptor for advanced glycation end-products; ROS, reactive oxygen species; TRPV4, transient receptor potential cation channel subfamily V member 4

It is now widely accepted that small, soluble hA oligomers elicit cytotoxicity in β-cells, whereas mature fibrils do not (Kayed et al., 2003; Zhang et al., 2008). Oligomers are said to form both inside and outside cells, but the exact cellular localization of the oligomerization processes are uncertain. Moreover, the precise molecular structure of these cytotoxic oligomers is also unknown. hA oligomers are proposed to directly cause β-cell damage through several mechanisms, which can be divided into two broad categories: membrane disruption and alteration of cellular pathways that lead to or cause apoptosis (see Figure 1 for a summary).

Mechanisms that may explain how hA oligomers can cause cellular and/or intracellular membrane disruption include channel formation, detergent-like disruption, and membrane remodeling (Rawat et al., 2018). hA also elicits activation of the c-Jun N-terminal kinase (JNK) pathway, the central mediator of apoptosis in β-cells (Subramanian et al., 2012), which could be through direct interaction with the Fas-associated death receptor on the cell surface (Zhang et al., 2008), or through indirect mechanisms such as generation of oxidative stress and inflammation (Subramanian et al., 2012).

Other pathways/processes that have been implicated in β-cell dysfunction include inflammation, oxidative stress, mitochondrial dysfunction, and endoplasmic reticulum (ER) stress (Baker et al., 2011; Volpe et al., 2018). These mechanisms are not mutually exclusive. It is possible that T2DM may not arise from a single pathogenic pathway but from a collection of perturbations to normal cellular processes. In fact, evidence suggests that hA oligomers may also play a role in these processes.

The process of amyloid formation induces reactive oxygen species (ROS) that may contribute to β-cell apoptosis over time (Zraika et al., 2009). Under low (nanomolar) hA concentrations for even short periods, hA aggregates increased NADPH oxidase (NOX)-1 expression via binding to the receptor for advanced glycation end-products (RAGE) (Borchi et al., 2013). Mitochondrial disruption is a major source of ROS and is also induced by hA, leading to caspase activation, release of cytochrome-c and apoptosis-inducing factor (AIF) into the cytosol, and eventually apoptosis (Li et al., 2011).

Extracellular application of hA to β-cells increases expression of heat shock proteins and signs of ER stress (Casas et al., 2007). Increased intracellular Ca^2+^ has also been reported, which may lead to ER stress and apoptosis via the caspase cascade (Casas et al., 2007; Casas et al., 2008). Elevated Ca^2+^ may be caused by the perturbation of the cell membrane evoked by hA and the activation of transient receptor potential cation channel subfamily V member 4 (TRPV4) channels (Casas et al., 2008). The interaction between hA and the cell membrane may also cause internalization of lipid-peptide complexes that localize to the ER, trigger the unfolded protein response, and modify insulin secretion and cell viability (Martinez-Navarro 2020).

hA oligomers can also induce release of pro-inflammatory cytokines. These cytokines, such as interleukin (IL)-1β, tumor necrosis factor α (TNF-α) and interferon γ (IFN-γ), impair insulin secretion by RIN-m5F β-cells, trigger ROS production, and cause mitochondrial dysfunction; impairing mitochondrial membrane potential and leading to apoptosis (Zhang et al., 2011). hA oligomers can trigger IL-1β processing in dendritic cells and macrophages, which triggers apoptosis in β-cells (Masters et al., 2010; Westwell-Roper et al., 2014). hA-induced elevation of IL-1β levels in islets is associated with upregulation of Fas, activation of caspase-8 and apoptosis in β-cells (Park et al., 2017). In the islets of hA-transgenic mice, hA-induced IL-1β secretion by pancreatic macrophages may contribute to β-cell death (Masters et al., 2010). Macrophage depletion in hA-transgenic mice inhibited inflammatory gene expression and improved glucose tolerance, while treatment with IL receptor agonist IL-1Ra improved β-cell function in T2DM patients (Westwell-Roper et al., 2014). Conversely, IL-1β stimulates islet β-cells to release increased amounts of hA, and impairs processing of pro-amylin, forming a positive feedback loop and potentially augmenting amyloid deposition (Park et al., 2017; Templin et al., 2020).

Intracellular and extracellular hA oligomers may primarily induce β-cell death through different mechanisms. In β-cells, intracellular hA aggregates evoked cytochrome c release from mitochondria, associated with caspase-9 activation and apoptosis (Wong et al., 2021). Blocking the Bax or caspase-9 pathway decreased hA-induced apoptosis. However, in islets cultured with extracellular hA, blocking the mitochondrial apoptosis pathway lowered hA-induced cytotoxicity only modestly. Fas inhibition was much more effective at rescuing cell viability, suggesting intracellular aggregates may trigger the mitochondrial apoptosis pathway and extracellular aggregates may trigger the Fas pathway.

To summarize, multiple mechanisms are implicated in hA-induced β-cell dysfunction and activation of apoptosis *in vitro*, including direct membrane disruption, receptor-mediated apoptosis and induction of cellular stress and inflammation, although it remains to be seen if all these play *in vivo* roles. Inflammation and oxidative stress are also involved in the development of insulin resistance in various tissues (Wellen & Hotamisligil, 2005). Polyphenols can target a broad range of these mechanisms and may therefore be a promising avenue for developing antidiabetic therapies.

## *In Vitro* Evidence for Inhibition of Amylin Aggregation and β-cell Protective Effects

The ability of various polyphenolic compounds to inhibit hA aggregation *in vitro* has been investigated extensively and are summarized in Table 1. Studied compounds include: resveratrol (derived from the skin of grapes and berries); epigallocatechin gallate (EGCG) and related green tea catechins; rutin and its aglycone quercetin (found in citrus fruits and buckwheat); curcumin (the principal flavonoid in turmeric); and oleuropein (from olive oil) amongst many others.

**TABLE 1:** Summary table of *in vitro* studies of polyphenolic compounds shown to inhibit human amylin (hA) fibril formation in biophysical studies.

Compound	Inhibits hA aggregation? (compound:hA molar ratio)	Ability to disaggregate fibrils? (compound:hA molar ratio)	Prevents hA-induced cytotoxicity? (compound:hA molar ratio)	Cell line used for cytotoxicity assay	References
Resveratrol	Yes (0.05:1–10:1)	Not studied	Yes (1:1)	INS-1E	Mishra et al. (2009)
	Yes (1:1 in INSE-1E cell culture)	Not studied	Yes (1:1)	INS-1E	Radovan et al. (2009)
	Yes (1:1–20:1)	No (10:1)	Not studied	-	Tu et al. (2015)
Epigallocatechin gallate	Yes (0.2:1–1:1)	Yes (1:1)	Yes (1:1)	INS-1	Meng et al. (2010)
	Yes (0.5:1–1:1)	Yes (1:2–5:1)	Not studied	-	Cao & Raleigh (2012)
	Yes (0.2:1–5:1)	Not studied	Not studied	-	Franko et al. (2018)
	Yes (20:1)	Not studied	Not studied	-	Chen, S. et al. (2020)
	Yes (2:1–20:1)	Yes (1:1–3:1)	Not studied	-	Wu et al. (2021)
Rutin	Yes (0.1:1–10:1)	Yes (6:1–11:1)	Not studied	-	Aitken et al. (2017)
	Yes (20:1)	Not studied	Not studied	-	Chen, S. et al. (2020)
Quercetin	Yes (2:1)	Not studied	Yes (small effect at 1:7.5, cytotoxic alone at high concentrations (IC_50_ = 110 µM))	RIN-m5F	Lopez et al. (2016)
	Yes (10:1)	Not studied	Not studied	-	Aitken et al. (2017)
Curcumin	Yes (0.01:1–1:1)	No (1:1)	Yes in INS 832/13 (0.67:1–1.67:1, cytotoxic alone at concentrations ≥25 µM).	INS 832/13, hA-transduced INS 832/13, HIP rat isolated pancreatic islets	Daval et al. (2010)
			No in INS 832/13 cells overexpressing hA and in isolated islets from HIP rats		
Baicalein	Yes (3:1–10:1)	Yes (3:1)	Yes (0.5:1–5:1)	INS-1	Velander et al. (2016)
Oleuropein	Yes (1:1–9:1)	Not studied	Yes (3:1–9:1)	RIN-m5F	Rigacci et al. (2010)
	Yes (1:1–20:1)	Yes (10:1)	Yes (1:1–10:1)	INS-1	Wu et al. (2017)
	Yes (0.67:1)	Not studied	Yes (2:1–20:1)	INS-1E	Chaari (2020)
Caffeic acid	Yes (0.4–4:1)	Not studied	Not studied	-	Sun et al. (2019)
	Yes (1:1)	Yes (1:1)	Yes (5:1)	Neuro2A	Wu et al. (2021)
Rosmarinic acid	Yes (0.04:1–4:1)	Not studied	Not studied	-	Sun et al. (2019)
	Yes (0.3:1–30:1)	Yes (1:1–3:1)	Yes (5:1)	INS-1, Neuro2A	Wu et al. (2021)
Morin	Yes (1:1–10:1)	Yes (1:1–5:1)	Not studied	-	Noor et al. (2012)

Amyloid formation is often measured by thioflavin T (ThT) fluorescence; however, some polyphenols have been shown to compete with ThT binding sites on hA fibrils (Daval et al., 2010). Therefore, a secondary technique such as circular dichroism spectroscopy, ion-mobility mass spectrometry, transmission electron microscopy, or atomic force microscopy should be used to confirm and validate the results of ThT measurements.

Compounds that have demonstrated hA aggregation-inhibitory properties *in vitro* include: resveratrol (Mishra et al., 2009; Tu et al., 2015); EGCG (Meng et al., 2010; Cao and Raleigh, 2012; Franko et al., 2018); rutin and quercetin (Aitken et al., 2017; Chen, S. et al., 2020); curcumin (Daval et al., 2010); baicalein (Velander et al., 2016), oleuropein (Rigacci et al., 2010; Wu et al., 2017); caffeic acid (Sun et al., 2019); and rosmarinic acid (Sun et al., 2019; Wu et al., 2021). Some, but not all, of these compounds have been shown to be capable of disaggregating pre-formed hA fibrils, including EGCG (Meng et al., 2010); rutin (Aitken et al., 2017); rosmarinic acid (Wu et al., 2021); baicalein (Velander et al., 2016), oleuropein (Wu et al., 2017), and morin (Noor et al., 2012).

It is important that the structures stabilized by polyphenolic compounds are not cytotoxic, lest treatment with these compounds could inadvertently accelerate β-cell death. Several studies show that the small aggregates stabilized by many polyphenols are “off-pathway” and non-cytotoxic. hA-EGCG complexes do not seed hA aggregation, suggesting these are off-pathway species (Meng et al., 2010). Oleuropein forms non-toxic structures with hA that are distinct from pure hA oligomers (Rigacci et al., 2010). Discrete molecular dynamics simulations suggest that resveratrol and curcumin also stabilize small, off-pathway hA oligomers (Nedumpully-Govindan et al., 2016). Both EGCG and rosmarinic acid can disaggregate pre-formed hA fibrils to amorphous, off-pathway aggregates. This disaggregation pathway was not the reverse of fibril formation and hA monomers were not formed (Wu et al., 2021).

Several polyphenols have protective effects against the cytotoxicity induced by hA treatment in pancreatic cell lines. Resveratrol prevented hA-evoked cytotoxicity in the INS-1E rat insulinoma cell line (Mishra et al., 2009; Radovan et al., 2009) and decreased oxidative stress in hA-overexpressing INS-1E cells (Hernandez et al., 2018). Similarly, EGCG prevented hA-induced toxicity in INS-1 cells (Meng et al., 2010), quercetin demonstrated a small effect in RIN-m5F rat insulinoma cells (Lopez et al., 2016), and curcumin in the INS 832/13 β-cell line (Daval et al., 2010). Oleuropein has shown a cytoprotective effect against hA in INS-1 (Wu et al., 2017; Chaari, 2020) and RIN-m5F cells (Rigacci et al., 2010). Rosmarinic acid formed nontoxic aggregates with hA that neutralized hA-induced cytotoxicity in INS-1 cells (Wu et al., 2021). Similarly, baicalein also prevented hA-induced cytotoxicity in INS-1 cells (Velander et al., 2016).

In summary, many natural polyphenols demonstrate the ability to inhibit amyloid formation by hA in in vitro tests, often forming off-pathway species and protecting β-cells from hA-evoked cytotoxicity.

## *In Vivo* Evidence of an Antidiabetic Effect of Polyphenols

Numerous studies in animal models have been done to test the antidiabetic effects of polyphenols, which are summarized in Table 2. Only a few of these have utilized a model expressing hA, the human, aggregating form of amylin. EGCG had some benefit in a study using hA-transgenic mice, where oral treatment for 3 weeks reportedly decreased pancreatic amyloid fibril levels in hemizygous mice but not in homozygous (Franko et al., 2018). Oral administration of rutin delayed diabetes onset and ameliorated disease severity and progression in hA-transgenic mice (Aitken et al., 2017). Similarly, in a study using transgenic HIP rats, 4-month dietary supplementation of rosmarinic acid reduced pancreatic amyloid deposition, serum hA oligomer levels and non-fasting blood glucose, and increased serum insulin levels (Wu et al., 2021).

**TABLE 2:** Summary table of *in vivo* studies of polyphenolic compounds in animal models of diabetes. Abbreviations: AMPK, AMP-activated protein kinase; CPT-1, carnitine palmitoyl transferase 1; eNOS, endothelial nitric oxide synthase; ER, endoplasmic reticulum; FFA, free fatty acids; GLUT-4, glucose transporter 4; hA, human amylin; HbA1c, hemoglobin A1c; HOMA-IR, Homeostatic Model Assessment of Insulin Resistance; NA, nicotinamide; NFκB, nuclear factor κ B; PECK, phosphoenolpyruvate carboxykinase; PGC-1α, peroxisome proliferator-activated receptor gamma coactivator 1α; PI3K, phosphoinositide 3-kinase; ROS, reactive oxygen species; SIRT1, sirtuin 1; SOD, superoxide dismutase; STZ, streptozotocin.

Compound	Animal model	Dose and route of administration	Duration	Findings	References
**Models expressing human amylin (hA)**					
Epigallocatechin gallate	hA-transgenic mice	0.4 mg/ml in drinking water	3 weeks	Decreased pancreatic amyloid in hemizygous mice but not in homozygous. Nonsignificant tendency to increase islet number in homozygous mice. No change in blood glucose, body weight or pancreatic insulin staining	Franko et al. (2018)
Rutin	hA-transgenic mice	0.5 mg/ml in drinking water	From weaning until death	Delayed diabetes onset, prolonged time to accelerated increases in blood glucose, fluid intake and body-weight loss	Aitken et al. (2017)
Rosmarinic acid	HIP rats	0.5% w/w dietary supplementation	4 months	Reduced pancreatic amyloid deposition, hA oligomer levels in sera, and non-fasting blood glucose. Prevented hypoinsulinemia	Wu et al. (2021)
**Non-hA expressing models of diabetes**					
Resveratrol	*db/db* mice	0.005 and 0.02% w/w dietary supplementation	6 weeks	Both doses decreased blood glucose levels. Higher dose decreased HbA1c levels and increased glucose tolerance, plasma insulin levels and pancreatic insulin staining.	Do et al. (2012)
				Higher dose increased hepatic glycolytic enzyme activity and decreased gluconeogenesis enzyme activity. Both doses increased skeletal muscle GLUT-4 protein. Both doses decreased plasma and hepatic lipid levels	
	*db/db* mice	20 mg/kg/day orally	12 weeks	No decrease in blood glucose levels but improved glucose tolerance and preserved islet β-cell mass. Decreased pancreatic ROS levels	Lee et al. (2012)
	NA-STZ and STZ-induced diabetic rats	0.05, 0.1, 0.5, 3.0, 6.0 and 10.0 mg/kg orally	Single dose or 3 mg/kg 3x daily for 7 days	Dose-dependently lowered plasma glucose (PI3K-dependent). Three and 10 mg/kg increased plasma insulin levels in NA-STZ rats but not STZ rats. Improved glucose tolerance in normal rats	Chi et al. (2007)
				In STZ rats, 3 mg/kg increased Akt phosphorylation in skeletal muscle (PI3K-dependent), while 7-day treatment increased GLUT-4 protein in skeletal muscle and decreased PEPCK protein in liver	
	NA-STZ and STZ-induced diabetic rats	5 mg/kg/day orally	30 days	Lowered fasting blood glucose and HbA1c levels, increased plasma insulin levels. Prevented β-cell degeneration.	Palsamy & Subramanian (2010)
				Normalized plasma levels of pro-inflammatory cytokines and antioxidants. Normalized pancreatic antioxidant enzyme activity. Decreased markers of oxidative stress in plasma and pancreas	
	STZ-induced diabetic rats	2.5 mg/kg/day orally	2 weeks	Lowered blood glucose levels. May increase glucose uptake in the heart via increased Akt, AMPK and eNOS phosphorylation, and GLUT-4 translocation	Penumathsa et al. (2008)
	Green tea extract	High-fructose diet-fed rats	0.5 g/100 ml drinking water	12 weeks	Prevented increased in fasting plasma glucose and insulin. Normalized glucose and insulin response. Lowered blood pressure.	Wu, Juan, Hwang et al. (2004)
					Normalized insulin-stimulated glucose uptake and GLUT-4 protein levels in adipocytes	
		Sprague Dawley rats	0.5 g/100 ml drinking water	12 weeks	No change in fasting plasma glucose after 4 and 6 weeks. After 12 weeks, reduced fasting plasma glucose and insulin levels and increased insulin sensitivity. Decreased plasma lipid levels. Increased glucose uptake in adipocytes	Wu, Juan, Ho et al. (2004)
		STZ mice and *db/db* mice	30, 150 or 300 mg/kg orally	Single dose	300 mg/kg lowered fasting blood glucose levels without altering insulin levels	Tsuneki et al. (2004)
Epigallocatechin gallate		STZ mice	100 mg/kg/day intraperitoneally	5 days with STZ then 5 days alone	Reduced hyperglycemia and partially preserved islet mass. Attenuated the induction of iNOS expression	Song et al. (2003)
		STZ rats	25 mg/kg/day orally	8 weeks	Decreased serum glucose and lipid levels. Decreased hepatic lipid peroxidation and attenuated the decrease in SOD activity	Roghani & Baluchnejadmojarad (2010)
	Sprague Dawley rats and Zucker rats	70–92 mg/kg/day intraperitoneally	7 days	Decreased serum glucose, insulin, leptin and lipid levels	Kao et al. (2000)
	*db/db* mice	1% w/w dietary supplementation	10 weeks	Decreased fasting blood glucose. No change in fasting plasma insulin. Improved glucose tolerance but no improvement in insulin sensitivity.	Ortsater et al. (2012)
				Decreased islet pathology. Reduced expression of ER stress markers in islets	
	*db/db* mice	0.25, 0.5, or 1% w/w dietary supplementation	7 weeks	Improved glucose tolerance. Dose-dependent reduction in blood glucose and increase in plasma insulin. Decreased plasma triacylglycerol.	Wolfram et al. (2006)
				Increased glucokinase expression, decreased PEPCK expression in liver. Increased acyl-CoA oxidase-1 and CPT-1 in liver and adipose	
	ZDF rats	0.5% w/w dietary supplementation	10 weeks	Improved glucose tolerance, decreased plasma FFA.	Wolfram et al. (2006)
	Non-obese diabetic (NOD) mice	0.05% w/v in drinking water	27 weeks	Lowered fasting blood glucose and HbA1c levels, improved glucose tolerance, and increased plasma insulin levels. Delayed onset of diabetes and reduced mortality rate of diabetic mice.	Fu et al. (2011)
				No effect on immune cell infiltrate in the pancreas. Increased plasma IL-10 and IL-12 levels but no effect on other measured cytokines	
	High fat diet-fed mice	50 mg/kg/day orally	4 weeks	No effect on fasting serum glucose, insulin or lipid levels but improved glucose tolerance	Zhang et al. (2021)
	Epicatechin	Alloxan-induced diabetic rats	30 mg/kg 2 x daily intraperitoneally	2 days prior to alloxan and 1 day after, or 4–5 days after	Prevented hyperglycemia, hypoinsulinemia, and preserved β-cell mass	Chakravarthy et al., 1981, Chakravarthy et al., 1982
		Alloxan-induced diabetic rats	100 mg/kg/day intraperitoneally	2 weeks	Treatment group tended towards lower blood glucose levels but not significant	Sheehan et al. (1983)
		STZ rats and BB/E rats	30 mg/kg 2 x daily intraperitoneally	6–9 days	No differences in blood glucose or β-cell mass in STZ rats.	Bone et al. (1985)
			Or 90 mg/kg/day orally		In diabetic BB/E rats, failed to prevent body weight loss or decrease plasma glucose. In prediabetic BB/E rats, failed to prevent diabetes onset and progression	
		Rutin	STZ rats	25, 50 or 100 mg/kg/day orally	45 days	All doses decreased fasting plasma glucose levels. 100 mg/kg increased plasma insulin and C-peptide, and decreased HbA1c levels. Decreased plasma oxidative stress markers and increased antioxidant levels	Kamalakkannan & Prince (2006)
			STZ rats	2, 25 or 50 mg/kg/day intraperitoneally	2 weeks	All doses decreased plasma glucose levels. Improved nerve function, decreased oxidative stress and inflammation in nerves, and normalized antioxidant enzyme activity	Tian et al. (2016)
	Fructose-fed rats		50 or 100 mg/kg/day orally	4 weeks	Decreased serum lipid levels. Higher dose improved renal function. Both doses decreased kidney and serum inflammation markers.	Hu et al. (2012)
Reversed hyperleptinemia and restored leptin and insulin signaling in kidney						
Quercetin	Fructose-fed rats		50 or 100 mg/kg/day orally	4 weeks	Decreased serum lipid levels. Improved renal function and decreased inflammation in kidney and serum. Reversed hyperleptinemia and restored leptin and insulin signaling in kidney	Hu et al. (2012)
	High cholesterol diet-fed rats		0.5% w/w dietary supplementation	4 weeks	Prevented an increase in plasma glucose, insulin and lipid levels, and pancreatic cholesterol content. Increased pancreatic insulin content.	Carrasco-Pozo et al. (2016)
		Prevented decreases in pancreatic ATP levels and antioxidant enzyme activity. Prevented increases in oxidative stress and inflammation. Increased expression of SIRT1 and PGC-1α				
	STZ rats	10 or 15 mg/kg/day intraperitoneally	10 days	Lowered plasma glucose and lipid levels, and improved glucose tolerance. Increased the number of pancreatic islets and increased hepatic hexokinase activity	Vessal et al. (2003)
	STZ rats	15 mg/kg/day intraperitoneally	4 weeks	Normalized serum glucose and insulin levels. Decreased pancreatic and serum oxidative stress markers and increased pancreatic antioxidant enzyme activity. Protected β-cells from degeneration	Coskun et al. (2005)
	STZ rats	50 mg/kg/day orally with or without 70 mg/kg/day sitagliptin	3 weeks	Decreased serum glucose and lipid levels, increased C-peptide levels. Reduced β-cell degeneration and increased insulin content. Decreased serum markers of oxidative stress and inflammation	Eitah et al. (2019)
	Alloxan-induced diabetic mice	20 mg/kg/day orally	3 weeks	Lowered fasting blood glucose. Increased glycolytic enzyme activity and decreased gluconeogenic enzyme activity in liver, skeletal muscle and kidney. Increased GLUT-4 expression in skeletal muscle, adipocytes and serum.	Alam et al. (2014)
				Increased antioxidant enzyme activity in pancreas, liver, kidney and skeletal muscle. Decreased markers of lipid peroxidation and liver and kidney dysfunction. Reduced DNA damage in pancreas, liver and kidney
	Curcumin	STZ rats fed a high-fat diet	50, 150 or 250 mg/kg/day orally	7 weeks	Decreased fasting blood glucose and plasma lipid levels. Increased insulin levels and insulin sensitivity. Increased phosphorylated AMPK in skeletal muscle	Na et al. (2011)
		Alloxan-induced diabetic rats	80 mg/kg/day orally	3 weeks	Lowered blood glucose and Hb1Ac levels. Reduced lipid peroxidation and increased antioxidant enzyme activity in plasma and liver	Arun & Nalini (2002)
		*db/db* mice	0.2 g/kg dietary supplementation	6 weeks	Decreased fasting blood glucose, Hb1Ac and plasma lipid levels, and increased insulin levels. Improved glucose tolerance and HOMA-IR.	Seo et al. (2008)
					Increased hepatic glucokinase activity and decreased gluconeogenic enzyme activity. Increased hepatic glycogen storage and lowered lipid peroxidation. Normalized hepatic lipid regulating and antioxidant enzyme activity. Increased lipoprotein lipase activity in skeletal muscle but not adipose	
		*db/db* mice	0.75% w/w dietary supplementation	8 weeks	No effect on blood glucose levels. Attenuated NFκB expression in liver. Increased hepatic AMPK expression but no effect on SIRT1 or PGC-1α. No effect on protein nitration	Jimenez-Flores et al. (2014)
High fat diet-fed mice		50 mg/kg/day orally	15 days	Decreased blood glucose and serum insulin levels. Improved HOMA-IR and glucose tolerance. Decreased lipid peroxidation levels in serum and skeletal muscle but not in adipose or liver. Reduced ROS in skeletal muscle	He et al. (2012)
Kaempferol		STZ rats fed a high-fat diet	50 or 150 mg/kg/day orally	10 weeks	High dose reduced fasting blood glucose. Both doses decreased serum insulin, lipid levels, and markers of hepatic dysfunction. Improved insulin resistance and decreased markers of inflammation in the liver and serum	Luo et al. (2015)
		STZ mice	50 mg/kg/day orally	12 weeks	Reduced fasting and non-fasting blood glucose levels, and incidence of overt diabetes. Improved glucose tolerance and plasma lipid profile. No effect on insulin levels.	Alkhalidy et al. (2018)
					Decreased hepatic glucose production, pyruvate carboxylase activity but increased glucokinase activity and glycogen storage. Increased glucose metabolism in skeletal muscle	
		*Caesalpinia bonduc* extract	Alloxan-induced diabetic rats	250 or 500 mg/kg intraperitoneally	8 weeks	Dose-dependent decrease in fasting blood glucose. Decreased serum insulin, leptin, amylin and peptide YY levels. Reduced β-cell degeneration.	Iftikhar et al. (2020)
Normalized hepatic glycolytic and gluconeogenic enzyme activity, and glycogen content. Increased hepatic and pancreatic antioxidant enzyme activity and decreased oxidative stress. Increased expression of components of the insulin signaling pathway							

Many studies have used diabetic models that do not recapitulate the amyloid component of the human disease, such as streptozotocin (STZ) models or leptin deficient *db/db* mice. Some of these models are more reminiscent of T1DM than T2DM. Nonetheless, they provide valuable information on some of the numerous antidiabetic effects that many polyphenolic compounds possess.

Resveratrol supplementation in *db/db* mice decreased blood glucose and HbA1c levels (Do et al., 2012), increased plasma insulin and pancreatic insulin content (Do et al., 2012), improved glucose tolerance and preserved islet β-cell mass (Lee et al., 2012). In nicotinamide (NA)-STZ-induced and STZ-induced diabetes in rats, oral resveratrol lowered blood glucose (Chi et al., 2007; Penumathsa et al., 2008; Palsamy and Subramanian, 2010). In NA-STZ-treated rats, resveratrol also increased insulin levels (Chi et al., 2007; Palsamy and Subramanian, 2010).

Green tea treatment prevented hyperglycemia, hyperinsulinemia and insulin resistance induced by a high-fructose diet in rats (Wu, Juan, Hwang, et al., 2004). It also lowered blood glucose levels in STZ-diabetic and *db*/*db* diabetic mice, although it did not alter serum insulin levels (Tsuneki et al., 2004). Green tea treatment had no effect on blood glucose after 4 or 6 weeks in normal rats, but lowered blood glucose and insulin levels after 12 weeks, as well as increasing insulin sensitivity (Wu, Juan, Ho, et al., 2004).

Individual green tea components have shown mixed results. Intraperitoneal administration of EGCG reduced hyperglycemia and partially preserved islet mass in STZ-treated mice (Song et al., 2003) and lowered blood glucose in both Sprague Dawley and Zucker rats (Kao et al., 2000). Oral EGCG had no blood glucose-lowering effect after 4 weeks in high fat diet (HFD)-fed mice (Zhang et al., 2021). However, longer-term oral administration has been reported to lower blood glucose in *db/db* mice (Wolfram et al., 2006; Ortsater et al., 2012), non-obese diabetic (NOD) mice (Fu et al., 2011), and STZ-rats (Roghani and Baluchnejadmojarad, 2010). EGCG supplementation also improved glucose tolerance (Wolfram et al., 2006; Ortsater et al., 2012; Zhang et al., 2021) and decreased islet pathology (Ortsater et al., 2012). Some studies reported increased insulin levels (Wolfram et al., 2006; Fu et al., 2011), while others reported a decrease (Kao et al., 2000).

Studies on epicatechin in alloxan-treated rats have also shown mixed results. Some have reported that it normalized blood glucose levels via β-cell regeneration (Chakravarthy et al., 1982) and had a protective effect when injected prior to alloxan treatment (Chakravarthy et al., 1981). However, others reported no effects (Sheehan et al., 1983; Bone et al., 1985).

In STZ-diabetic rats, oral rutin treatment decreased fasting plasma glucose levels (Kamalakkannan and Prince, 2006; Tian et al., 2016) and increased insulin and C-peptide levels (Kamalakkannan and Prince, 2006). Both rutin and its aglycone quercetin restored impaired insulin and leptin signaling pathways in fructose-fed rats (Hu et al., 2012).

Quercetin has shown similar effects to rutin. Oral quercetin supplementation in alloxan-induced diabetic mice (Alam et al., 2014), STZ rats (Eitah et al., 2019) and rats fed a high-cholesterol diet (Carrasco-Pozo et al., 2016), as well as intraperitoneal quercetin administration to STZ-rats (Vessal et al., 2003; Coskun et al., 2005), lowered plasma glucose levels. Quercetin also increased insulin levels and protected β-cells from STZ-induced degeneration (Vessal et al., 2003; Coskun et al., 2005; Eitah et al., 2019). In rats receiving a high-cholesterol diet, quercetin prevented an increase in insulin levels (Carrasco-Pozo et al., 2016).

Oral administration of curcumin to rats fed both a HFD and treated with STZ, led to lower fasting blood glucose levels, increased insulin levels and insulin sensitivity (Na et al., 2011). Curcumin treatment lowered blood glucose and Hb1Ac levels in alloxan-induced diabetic rats (Arun and Nalini, 2002) and *db*/*db* mice (Seo et al., 2008). Curcumin was also reported to improve insulin resistance and glucose tolerance in *db*/*db* mice (Seo et al., 2008) and HFD-fed mice (He et al., 2012), and also increased insulin levels in *db*/*db* mice (Seo et al., 2008). By contrast, in a similar study of *db*/*db* mice, curcumin supplementation had no effect on blood glucose levels (Jimenez-Flores et al., 2014).

A study using *Caesalpinia bonduc* (CPP) extract (containing high levels of polyphenols, including gallic acid, caffeic acid, *p*-coumaric acid, chlorogenic acid, protocatechuic acid, and epicatechin) administered to alloxan-induced diabetic rats found that it restored serum glucose and insulin levels toward normal and reduced β-cell degeneration (Iftikhar et al., 2020). Kaempferol treatment in HFD- and STZ-treated rats reduced circulating blood glucose, insulin levels and insulin resistance (Luo et al., 2015). Another study using STZ-mice found that kaempferol decreased fasting and non-fasting glucose levels, the incidence of overt diabetes and improved glucose tolerance. However, no effect on insulin levels was found (Alkhalidy et al., 2018).

To summarize, several polyphenols have shown promising antidiabetic effects following oral administration. Many demonstrate a blood glucose-lowering effect while some also increased circulating insulin levels (likely due to a protective effect on pancreatic β-cells), whereas others decreased insulin (perhaps due to increased insulin sensitivity). Care must be taken in interpreting the results from diabetic animal models. In some cases, such as in STZ- or alloxan-induced diabetes, the beneficial effects of polyphenol treatment may relate to the specific method of diabetes induction (for example, inflammation).

## Clinical Evidence for an Antidiabetic Effect of Polyphenols

While evidence for antidiabetic effects in non-clinical models appears to be promising, that from human studies is more limited and ambiguous. These are summarized in Table 3. The effects of polyphenol intervention in human randomized controlled trials have recently been reviewed in a meta-analysis by Raimundo et al. (2020). Briefly, the authors found there was evidence that polyphenol consumption lowered fasting blood glucose levels overall but did not affect insulin levels or Homeostatic Model Assessment of Insulin Resistance (HOMA-IR) values. The effect of polyphenol intervention was strongest in patients with diagnosed diabetes and greater when used in conjunction with antidiabetic medication.

**TABLE 3:** Summary table of studies of the antidiabetic effects of polyphenolic compounds in humans. Abbreviations: AMPK, AMP-activated protein kinase; BMI, body mass index; GIP, gastric inhibitory polypeptide; GLP-1, glucagon-like peptide 1; GSK-3β, glycogen synthase kinase-3β; HbA1c, hemoglobin A1c; HOMA-β, Homeostatic Model Assessment of β-cell Function; HOMA-IR, Homeostatic Model Assessment of Insulin Resistance; PGC-1α, peroxisome proliferator-activated receptor gamma coactivator 1α; RCT, randomized controlled trial; SIRT1, sirtuin 1; T2DM, type 2 diabetes mellitus.

Compound	Type of study	Dose per day (oral)	Duration	Participant criteria	Outcomes	References
Polyphenols (general)	Meta-analysis	33–2093 mg	4 weeks–1 year	Diagnosed T2DM (taking or not taking medication), at high risk of T2DM, or low risk of T2DM	Reduced fasting blood glucose levels and small decrease in HbA1c levels. No effect on insulin levels or HOMA-IR.	Raimundo et al. (2020)
					Effect was strongest in patients with diagnosed T2DM, especially in conjunction with antidiabetic medication	
Green tea catechins	Meta-analysis	208–1,207 mg	2 weeks–6 months	Diagnosed or borderline T2DM, overweight to obese subjects, or healthy subjects	Decreased fasting blood glucose and HbA1c levels. No effect on fasting insulin levels or HOMA-IR.	Liu et al. (2013)
					Effect only seen in studies with catechin intake ≥457 mg (median), and only in subjects with/at risk of metabolic syndrome. No difference between short or long duration studies	
	Meta-analysis	235.64–1,206.9 mg	3–24 weeks	Elevated fasting blood glucose, overweight to obese subjects, or healthy subjects	Lowered fasting blood glucose. No change in HbA1c, insulin levels or HOMA-IR.	Zheng et al. (2013)
					Effect only seen in studies with a duration ≥12 weeks. Dose and health status were not effect modifiers	
	Meta-analysis	200 mg polyphenols – 4 cups green tea	4 weeks–18 months	Participants with T2DM or prediabetes	No effect on fasting blood glucose, insulin, HbA1c or HOMA-IR.	Yu et al. (2017)
	Epigallocatechin gallate	RCT	300 mg	8 weeks	Diagnosed T2DM and not receiving insulin treatment (*n* = 44)	Decreased fasting blood glucose and marker of inflammation. No effect on insulin levels or HOMA-IR.	Hadi et al. (2020)
		RCT	800 mg	8 weeks	Overweight or obese subjects (*n* = 88)	No change in fasting glucose, HbA1c, insulin, lipid levels, glucose tolerance, HOMA-IR or HOMA-β. There was a decrease in diastolic blood pressure	Brown et al. (2009)
Resveratrol		RCT	10 mg	4 weeks	Diagnosed T2DM and not receiving insulin treatment (*n* = 19)	Extended time to maximal tissue glucose levels after a meal and decreased HOMA-IR. Did not affect blood glucose, serum insulin, amylin, GLP-1, GIP or lipid levels, or HOMA-β. Decreased marker of oxidative stress and increased marker of insulin signaling	Brasnyo et al. (2011)
	RCT crossover	150 mg	30 days	Participants with obesity (*n* = 11)	Decreased plasma glucose, insulin, leptin, triglycerides levels, and HOMA-IR. Delayed peak glucose and insulin responses after a meal. Decreased systolic blood pressure. Decreased inflammatory markers. Increased markers of mitochondrial oxidative metabolism (including AMPK, SIRT1 and PGC-1α) and improved liver function	Timmers et al. (2011)
	RCT	40 or 500 mg	6 months	Diagnosed T2DM and not receiving insulin treatment (*n* = 179)	No change in fasting serum glucose, HbA1c, insulin or lipid levels, liver function biomarkers, HOMA-IR or blood pressure. Decrease in C-reactive protein (inflammation)	Bo et al. (2016)
	RCT crossover	1,000 mg	5 weeks	Diagnosed T2DM managed by diet only (*n* = 14)	No change in fasting or post-prandial blood glucose or GLP-1 levels. No effect on HbA1c levels	Thazhath et al. (2016)
	RCT	1,500 mg	4 weeks	Participants with obesity (*n* = 24)	No effect on fasting plasma glucose, HbA1c, insulin, lipid levels, HOMA-IR or blood pressure. No effect on insulin sensitivity by hyperinsulinemic euglycemic clamp. No effect on inflammatory or liver biomarkers, or AMPK and SIRT1 activity	Poulsen et al. (2013)
	RCT	3,000 mg	12 weeks	Diagnosed T2DM, on oral hypoglycemic treatment (*n* = 10)	No change in fasting plasma glucose, insulin or lipid levels, or in HOMA-IR. Nonsignificant decrease in HbA1c. Did increased skeletal muscle SIRT1 expression, pAMPK:AMPK ratio, and resting metabolic rate	Goh et al. (2014)
	Grape extract/Grape extract + resveratrol	RCT	302 mg polyphenols ±16.2 mg resveratrol	12 months	Participants with T2DM, stable coronary heart disease and hypertension taking medication (*n* = 35)	No effect on serum glucose, HbA1c, lipid levels, or blood pressure. Several inflammation markers were decreased	Tome-Carneiro et al. (2013)
	Curcumin	RCT	180 mg	12 weeks	At risk of developing T2DM and BMI ≥25 (*n* = 29)	No change in fasting blood glucose but lowered serum insulin levels and HOMA-IR. Decreased serum amylin and GSK-3β levels	Thota et al. (2020)
		RCT	300 mg (curcuminoids)	3 months	Diagnosed T2DM and BMI ≥24, on oral hypoglycemic and/or insulin treatment (*n* = 100)	Decreased fasting serum glucose, HbA1c, lipid levels, and HOMA-IR. No effect on liver function biomarkers	Na et al. (2013)
		RCT phase 2	500 mg (≥95% curcuminoids, ≥65% curcumin)±30 mg zinc	3 months	Overweight or obese subjects with prediabetes (*n* = 82)	Curcumin with and without zinc decreased fasting plasma glucose, postprandial glucose, serum insulin, HbA1c and insulin resistance. Increased insulin sensitivity. Curcumin + zinc group only decreased BMI.	Karandish et al. (2021)
		RCT	1,500 mg (curcuminoids)	9 months	Participants with prediabetes not taking medication (*n* = 240)	Decreased the risk of developing T2DM. Reduced fasting blood glucose, HbA1c, and C-peptide levels. Improved glucose tolerance, HOMA-IR and HOMA-β	Chuengsamarn et al. (2012)
Quercetin		RCT	250 mg	8 weeks	Diagnosed T2DM and not receiving insulin treatment (*n* = 47)	No effect on fasting blood glucose, HbA1c levels, serum insulin, lipid levels, or HOMA-IR. Increased serum antioxidant capacity and decreased markers of oxidative stress	Mazloom et al. (2014)
		Meta-analysis	100–1,000 mg	4–12 weeks	Subjects with (pre)hypertension, polycystic ovary syndrome or T2DM, or were overweight or obese	Overall, no significant effect on fasting plasma glucose, serum insulin, HbA1c or HOMA-IR. Did decrease fasting glucose in studies ≥8 weeks and using ≥500 mg doses. Also decreased insulin levels in studies with participants <45 years and doses ≥500 mg	Ostadmohammadi et al. (2019)
Olive leaf extract/Oleuropein	RCT crossover	51.1 mg oleuropein, 9.7 mg hydroxytyrosol	12 weeks	Males with BMI between 25 and 30 (*n* = 45)	Improved glucose tolerance, insulin sensitivity and β-cell function. Increased IL-6 but no change in other inflammatory markers. No effect on lipid levels or liver function biomarkers	de Bock et al. (2013)
	RCT crossover	136.2 mg oleuropein, 6.4 mg hydroxytyrosol	6 weeks	Males with prehypertension (*n* = 60)	No change in fasting glucose, insulin or HOMA-IR. Decreased blood pressure and plasma lipid levels. Reduced IL-8 but no effect on other inflammatory markers	Lockyer et al. (2017)
	RCT	320.8 mg oleuropein, 11.9 mg hydroxytyrosol	12 weeks	Participants with prediabetes and BMI between 23 and 29.9 (*n* = 56)	Decreased fasting plasma glucose and lipid levels, however increased HbA1c was observed. No change in insulin levels or HOMA-IR.	Araki et al. (2019)
	RCT	500 mg olive leaf extract	14 weeks	Diagnosed T2DM and not receiving insulin treatment (*n* = 79)	No change in fasting or postprandial blood glucose but decreased HbA1c and fasting insulin levels	Wainstein et al. (2012)

A great number of trials have been performed using green tea and related catechins, but results have been inconsistent. A meta-analysis of randomized controlled trials using green tea or extract concluded that green tea catechins significantly lowered fasting blood glucose and hemoglobin A1c (HbA1c) levels (Liu et al., 2013). Subgroup analysis suggested that the blood glucose-lowering effect was only seen in those with higher catechin intakes, and only in subjects at risk of metabolic syndrome but not in healthy individuals. Similarly, another meta-analysis reported that catechins significantly lowered fasting blood glucose, but no significant changes in insulin levels, HbA1c or HOMA-IR were reported (however, fewer studies have reported results for these outcomes) (Zheng et al., 2013). Subgroup analysis indicated that a study duration of at least 12 weeks was needed for the effect on fasting blood glucose to be seen. By contrast, a more recent meta-analysis examining the effect of green tea on patients with T2DM or prediabetes found no effect on fasting blood glucose, HbA1c, insulin or HOMA-IR. However, the small sizes and varying quality of the studies examined may mean these findings are less reliable (Yu et al., 2017).

Studies on the major green tea polyphenol EGCG have been similarly contrary. A 300 mg/day dose in subjects with diabetes resulted in reduced fasting blood glucose levels, although there was no change in insulin levels or HOMA-IR (Hadi et al., 2020). However, an 800 mg/day dose for the same duration in obese participants failed to show any effect on fasting blood glucose, HbA1c, insulin, glucose tolerance or HOMA-IR (Brown et al., 2009).

Results from human studies on resveratrol have also been conflicting. Four-weeks supplementation of a low dose (10 mg/day) of resveratrol decreased HOMA-IR in T2DM patients but did not affect serum insulin levels or indexes of β-cell function (Brasnyo et al., 2011). By contrast, 12 weeks of a much higher dose (3 g/day) in subjects with T2DM did not show an improvement in HOMA-IR, although there was a nonsignificant decrease in HbA1c (Goh et al., 2014). Similarly, a 5-weeks intervention with 1 g/day of resveratrol found no change in fasting or post-prandial blood glucose or HbA1c (Thazhath et al., 2016). A study in obese participants found no effect of 1,500 mg/day resveratrol over 4 weeks on insulin resistance, fasting glucose, or insulin (Poulsen et al., 2013). This contrasts with a study by Timmers et al. which found that 30 days of 150 mg/day resveratrol reduced fasting plasma glucose, insulin, triglycerides, and HOMA-IR (Timmers et al., 2011). Trials with longer follow-up periods have also failed to demonstrate significant effects. A 6-months trial in T2DM patients did not lead to any change in serum glucose, HbA1c, insulin, C-peptide, HOMA-IR or FFA (Bo et al., 2016). Likewise, low-dose resveratrol for 12 months in patients with T2DM and hypertension failed to find any effect in serum glucose, HbA1c or lipids, although several inflammation markers were altered (Tome-Carneiro et al., 2013).

Trials using curcumin supplementation have generally reported more positive results. Twelve weeks of curcumin treatment in subjects with a high risk of developing T2DM lowered serum insulin levels and insulin resistance, but not fasting blood glucose (Thota et al., 2020). Curcumin treatment also decreased serum glycogen synthase kinase-3β (GSK-3β) and hA levels (overexpression of both is linked to T2DM). A 9-months trial in patients with prediabetes found that supplementation with curcuminoid (a mixture of curcumin analogs) decreased the risk of developing T2DM and improved HOMA-IR, β-cell function (HOMA-β), and C-peptide levels (Chuengsamarn et al., 2012). In patients with either diagnosed T2DM or prediabetes, curcuminoid treatment for 3 months resulted in decreased fasting blood glucose, HbA1c levels and insulin resistance (Na et al., 2013; Karandish et al., 2021). Furthermore, FFA and triglyceride levels were reduced in patients with diabetes (Na et al., 2013) and serum insulin decreased in prediabetic subjects (Karandish et al., 2021).

Few human trials using quercetin have been conducted in patients with T2DM. Quercetin supplementation (250 mg/day) for 8 weeks in diabetic patients increased serum total antioxidant capacity, but did not improve fasting blood glucose, HbA1c levels, insulin levels, insulin sensitivity or blood lipid profile (Mazloom et al., 2014). A meta-analysis of studies on quercetin’s effect on glycemic parameters in different population groups also found that there was no significant effect on fasting blood glucose, insulin, HbA1c or HOMA-IR. However, it did decrease fasting glucose in studies ≥8 weeks and using ≥500 mg doses. Additionally, quercetin decreased insulin levels in studies with participants <45 years and doses ≥500 mg (Ostadmohammadi et al., 2019).

A range of studies have been performed using olive leaf extract, the major component of which is oleuropein. Olive leaf extract (321 mg/day oleuropein) decreased fasting plasma glucose in prediabetic participants, although interestingly an increase in HbA1c was observed. There was no change in insulin or HOMA-IR (Araki et al., 2019). However, 500 mg/day of olive leaf extract patients with T2DM resulted in no change in fasting or postprandial blood glucose (although HbA1c and insulin levels were lowered) (Wainstein et al., 2012). Similarly, there was no effect of 136 mg/day oleuropein on fasting blood glucose, insulin or HOMA-IR in males with prehypertension (Lockyer et al., 2017). However, a lower dose of oleuropein (51 mg/day) did improved glucose tolerance and insulin sensitivity in overweight males (de Bock et al., 2013).

In summary, despite the considerable attention and number of randomized control trials involving the effect of polyphenols on metabolic or diabetes-related outcomes, results remain controversial. Conflicting results may be due to differences in the dose and duration of study, health status of participants, whether participants were taking other medication, and the limited sample sizes often involved. It should be noted that results are not necessarily consistent with a dose-dependent nor time-dependent effect of the intervention. In general, evidence suggests that polyphenol interventions show some promise in lowering blood glucose and perhaps improving insulin sensitivity, but more and better studies are required. Natural polyphenols have a good safety profile and are consumed in relatively high quantities in a regular human diet (Sequeira & Poppitt, 2017). Few adverse events have been reported in clinical studies. One should note that pancreatic amyloid cannot be measured in live subjects and that serum levels of hA or proamylin are often not measured as a biomarker, despite evidence that suggests high levels may be related to amyloid deposit and are associated with insulin resistance and T2DM (Zheng et al., 2010).

## Potential Mechanisms of Action

The myriad health-promoting effects of polyphenols can be attributed to their structural similarities and potential to modulate several cellular pathways common to many diseases, including diabetes. Many mechanisms of action may be responsible for these effects, including the ability to inhibit amyloid formation, scavenge ROS, increase cellular antioxidant capability, inhibit inflammation and cellular stress, ultimately inhibiting caspase activation and apoptosis (Wang et al., 2001; Carrasco-Pozo et al., 2016). Polyphenolic compounds may also regulate insulin signaling, glucose metabolism, lipid levels and other pathways. Together, these mechanisms may underlie the β-cell protective, insulin sensitizing and hypoglycemic effects of polyphenols.

### Inhibition of Human Amylin Aggregation

#### Structure-activity Relationships

**FIGURE 2:**
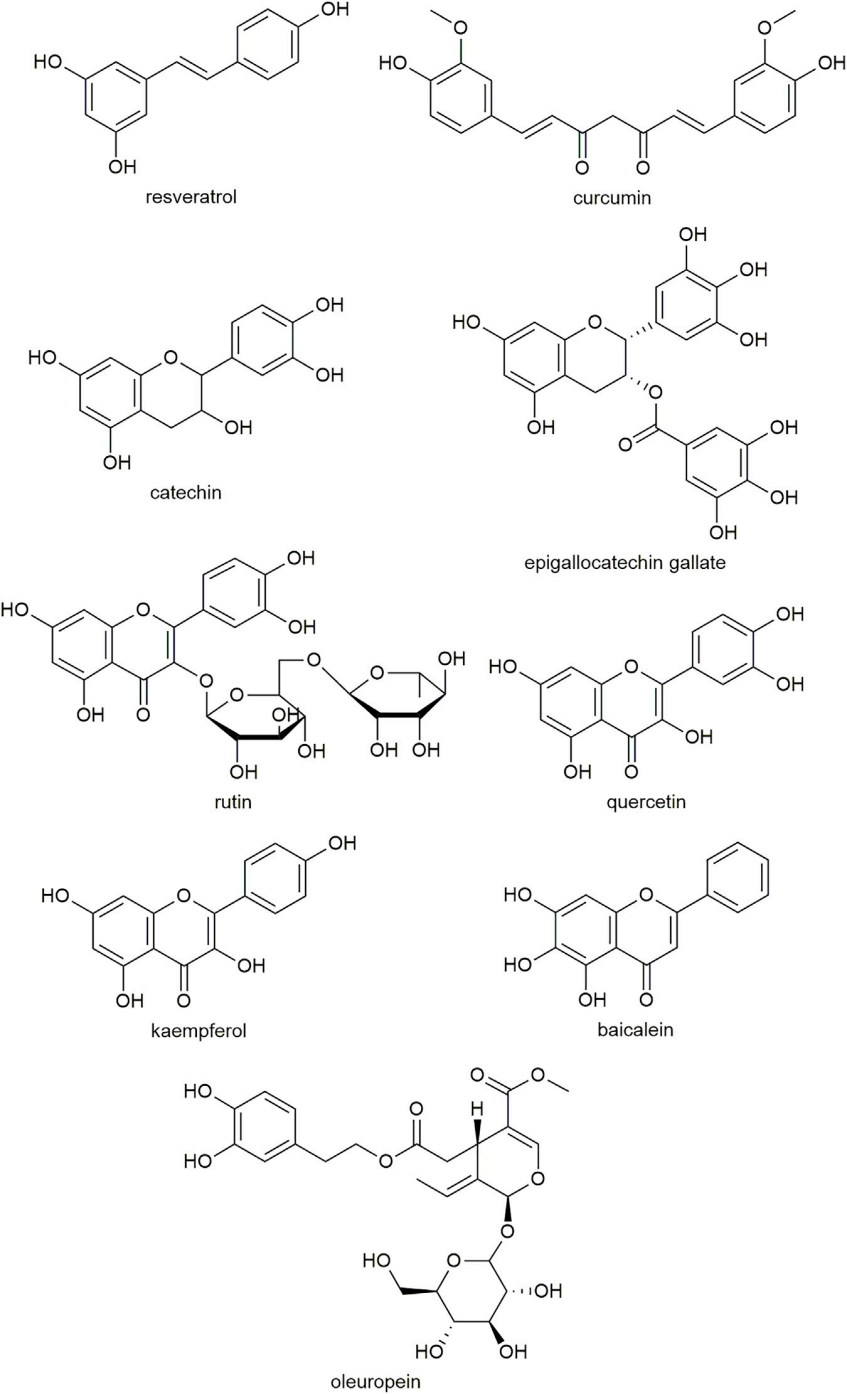
Examples of the molecular structure of polyphenolic compounds that have been studied for antidiabetic activity.

Polyphenolic compounds that are effective at inhibiting amyloid formation have several structural similarities (Figure 2). These include the presence of at least two phenolic rings, with two to six atom linkers between them and at least two hydroxyl groups (Porat et al., 2006). These structural similarities, as well as the promiscuity of polyphenolic compounds to inhibit amyloid formed from various peptides, including hA, amyloid-β and α-synuclein, suggest that the structural conformation is important for interacting with the aromatic residues and β-sheet structures common to amyloids (Porat et al., 2006).

Studies have found that polyphenolic compounds require at least two vicinal hydroxyl groups (such as a catechol moiety) to retain amyloid inhibitory activity and prevent hA-induced cytotoxicity (Velander et al., 2016; Wu et al., 2017). Replacing hydroxyl groups with methoxy groups causes the compound to lose activity, suggesting the hydroxyl groups may be involved in the interaction with hA (Velander et al., 2016). Furthermore, the flavonoid scaffold lacking any hydroxyl groups can bind with hA but does not inhibit fibrillation, suggesting the flavonoid core structure may interact with the hA peptide but that hydroxyl groups are required to prevent aggregation (Chen, W. L. et al., 2020).

The catechol moiety alone shows inhibitory effects against amyloid-β fibrillation (Huong et al., 2010). However, it has lower activity than larger molecules such as dopamine and l-DOPA. Additional ring structures on a catechol may make the compound more hydrophobic, increasing its binding affinity with amyloid structures (Porat et al., 2006). The side chains on the A and C rings in polyphenols also play a role in binding. Compounds containing two catechol moieties appear to be more effective at both inhibition of hA aggregation and disaggregation than analogues comprising only one catechol moiety (Nomoto et al., 2021). For example, the efficacy of rosmarinic acid was similar to the additive effects of its two substituents, caffeic acid and salvianic acid, on the inhibition of fibril formation and hA-induced cytotoxicity (Wu et al., 2021).

Due to the difficulty in observing the early stages of hA oligomer formation, the exact binding mechanism of many polyphenols is unclear. Despite the structural similarities, different polyphenols may interact with hA in different ways, and different hypotheses have been proposed to explain the binding interactions.

#### Covalent Adducts

One hypothesis is that the catechol moiety common to many polyphenolic compounds forms covalent adducts with amyloidogenic peptides, involving autoxidation of the hydroxyl groups to an o-quinone intermediate that conjugates with the peptide (Sato et al., 2013; Velander et al., 2016). Vicinal hydroxyl groups are readily oxidized to form an o-quinone, which can form bonds with nucleophilic residues such as Cys, Lys and Arg (Sato et al., 2013; Velander et al., 2016). The presence of three contiguous hydroxyl groups may facilitate even greater autoxidation (Akaishi et al., 2008; Cao and Raleigh, 2012; Ushikubo et al., 2012; Sato et al., 2013). Baicalein (Velander et al., 2016) (+)-taxifolin (Sato et al., 2013), and caffeic acid (Sun et al., 2019) are suggested to form covalent adducts with hA via an o-quinone intermediate.

#### π-π Stacking

Not all molecules that inhibit amyloid formation contain a catechol-like structure and studies with some polyphenols (resveratrol, curcumin, EGCG) indicate covalent binding may not be necessary (Sato et al., 2013; Tu et al., 2015). Amyloidogenic proteins have an abundance of aromatic residues and π-π stacking is considered to play a crucial role in protein self-assembly. hA contains three aromatic residues: Phe15, Phe23, and Tyr37 (Cooper et al., 1989). Phenol rings can interact with the benzene rings of aromatic residues via π-π stacking; however, they have a different stacking mechanism that prevents fibril formation (Porat et al., 2006). Additional structures are needed to form stabilizing interactions, such as hydrophobic interactions, hydrogen bonding, π-cation interactions between aromatic and cationic (Lys or Arg) sidechains, carbon-hydrogen bonding, van der Waals, alkyl, and π-alkyl interactions (Mo et al., 2016; Franko et al., 2018). EGCG (Mo et al., 2016) and resveratrol (Jiang et al., 2011) are proposed to inhibit hA amyloid formation by interfering with aromatic stacking.

#### Hydrophobic Core

Even for compounds that interact with hA via covalent adducts or π-π stacking, hydrophobic interactions play a large role in stabilizing the interaction of the polyphenol to the hA peptide. Another postulated mechanism of polyphenol-peptide binding is that multiple polyphenol molecules form a hydrophobic core to which hA peptides bind, forming off-pathway complexes stabilized by π- π stacking, as well as hydrogen bonds between the hydroxyl groups and the peptide side chains or backbones (Nedumpully-Govindan et al., 2016).

### Antioxidant Activity

Polyphenols are renowned for their antioxidant activity and ability to protect cells from oxidative stress. Several studies indicate that this may contribute to the antidiabetic effects of polyphenolic compounds. Mechanisms of action underlying this activity include ROS scavenging, reducing lipid peroxidation, increasing antioxidant enzyme activity, inhibiting the mitogen-activated protein kinase (MAPK)/JNK pathway that mediates apoptosis in β-cells (Iftikhar et al., 2020), and chelating metal ions. Polyphenols can also protect against oxidative damage by inhibiting inflammation, mitochondrial dysfunction, and ER stress (discussed in later sections).

Resveratrol has been reported to decrease levels of oxidative stress markers such as TSC2 nitration and 8-hydroxydeoxyguanosine (Lee et al., 2012; Hernandez et al., 2018), and preserve β-cell mass in *db/db* mice (Lee et al., 2012). In NA-STZ-induced diabetic rats, resveratrol decreased levels of lipid peroxides, hydroperoxides and protein carbonyls in both plasma and pancreatic tissue (Palsamy and Subramanian, 2010). In the plasma, resveratrol increased the levels antioxidants vitamin C, vitamin E, glutathione and ceruloplasmin. In the pancreas, activities of the antioxidant enzymes superoxide dismutase (SOD), catalase, glutathione peroxidase (GPx) and glutathione S-transferase (GST) were increased. Imaging of the pancreas showed that resveratrol had a β-cell protective effect.

EGCG and quercetin both lower lipid peroxidation in STZ-rats and rescue SOD activity (Coskun et al., 2005; Roghani and Baluchnejadmojarad, 2010; Eitah et al., 2019). Quercetin also reduced the STZ-induced increase in serum nitric oxide levels, rescued GPx and catalase activities, and protected β-cells from degeneration (Coskun et al., 2005). Similarly, quercetin recovered the activities of SOD, catalase, GST, and sulfhydryl levels in alloxan-induced diabetic mice (Alam et al., 2014). Quercetin treatment can also decrease levels of cholesterol-induced ROS, superoxide radicals and lipid peroxidation, and prevent cholesterol-induced deceases in SOD and GPx activity (Carrasco-Pozo et al., 2016).

In STZ-rats, rutin decreased levels of ROS (Tian et al., 2016) and lipid peroxidation (Kamalakkannan and Prince, 2006), increased activities of SOD, GPx, catalase and GST (Tian et al., 2016), increased plasma levels of antioxidants (Kamalakkannan and Prince, 2006), and preserved islet function (Kamalakkannan and Prince, 2006). Rutin can suppress high-glucose-stimulated NOX-4 expression, ROS production, and nod-like receptor protein 3 (NLRP3), caspase-1 and IL-1β protein levels (Wang et al., 2017). It also attenuated lipoteichoic acid-induced ROS generation and MAPK activation, decreased cyclooxygenase-2 protein levels, and increased SOD, catalase and GPx protein expression (Gutierrez-Venegas et al., 2020).

Likewise, CPP extract reduced ROS and lipid peroxidation in rats with alloxan-induced diabetes, and increased the activities of catalase, SOD and GPx (Iftikhar et al., 2020). It also decreased MAPK-8/JNK-1 phosphorylation and JNK pathway activation in the β-cells of these rats.

In the liver and plasma of rats with alloxan-induced diabetes, curcumin reduced lipid peroxidation, elevated the concentration of glutathione and increased the activity of GPx, possibly by reducing the level of glucose flux into the polyol pathway (Arun and Nalini, 2002). Curcumin and its analogues induced the expression of antioxidant enzymes heme oxygenase 1, the regulatory subunit of γ-glutamyl-cysteine ligase, and NAD(P)H:quinone oxidoreductase 1 in human islets and increased the glutathione content (Balamurugan et al., 2009). Curcumin attenuated a HFD-induced decrease in level of transcription nuclear factor erythroid-2-related factor-2, which regulates the expression of several antioxidant enzymes, and its downstream target heme oxygenase 1 in skeletal muscle (He et al., 2012). It also decreased lipid peroxidation in the serum and skeletal muscle of HFD-fed mice (He et al., 2012), and in the erythrocytes and liver of *db*/*db* mice (Seo et al., 2008).

Metal ions also play a role in the generation of ROS by participating in redox reactions. The catechol moiety present on many polyphenols is known for its ability to chelate metal ions such as Fe^2+^ and Cu^2+^, thus decreasing ROS generation and lipid peroxidation (Perron and Brumaghim, 2009). Quercetin, rutin, catechin, EGCG, curcumin, baicalein, myricetin and others have all demonstrated this property (Afanas’ev et al., 1989; Perron and Brumaghim, 2009).

The antioxidant activity of polyphenols alone cannot explain their protective effect against amyloid-induced cytotoxicity. Resveratrol and catechin together have a synergistic effect against amyloid-β-induced cytotoxicity, whereas no synergy was observed against hydrogen peroxide-induced cytotoxicity (Conte et al., 2003). The antioxidant activity of a compound does not correlate well to its ability to protect against amyloid-β-induced cytotoxicity, although it can modulate it (Wang et al., 2001).

It should be noted that β-cell preservation in STZ- or alloxan-treated animals following polyphenol treatment may be related to the specific mechanism of cytotoxicity caused by these compounds, which is inhibited by antioxidant activity (Chakravarthy et al., 1981; Vessal et al., 2003). Generally, STZ and alloxan-treated animals better recapitulate the pathogenesis of T1DM than T2DM diabetes, although oxidative stress and inflammatory cytokines are also a feature of T2DM.

### Anti-Inflammatory Activity

Another potential component of the antidiabetic effect of polyphenols is their apparent ability to modulate inflammation. Polyphenols have been reported to prevent cytokine-induced cellular damage, decrease levels of pro-inflammatory cytokines, increase levels of anti-inflammatory cytokines, and inhibit nuclear factor κ B (NFκB), the major transcription factor that promotes inflammation (Baker et al., 2011).

EGCG inhibits cytokine-induced (IL-1β, IFN-γ or TNF-α) cell death in isolated mouse islet cells (Song et al., 2003), RIN-m5F insulinoma cells (Han, 2003; Zhang et al., 2011), and cultured human islets (Fu et al., 2011). This may be achieved by inhibiting the NFκB pathway and expression of inducible nitric oxide synthase (iNOS) (Han, 2003), and caspase-3 activity (Fu et al., 2011). Intraperitoneal treatment of STZ-treated mice with EGCG protected mice from diabetes by inhibiting iNOS expression (Song et al., 2003). EGCG prevented cytokine-induced generation of ROS, restored insulin secretion, and preserved mitochondrial function in RIN-m5F cells (Zhang et al., 2011).

Both rutin and quercetin lowered renal levels of inflammatory cytokines IL-1β, IL-6, IL-18 and TNF-α in fructose-fed rats, likely mediated by the associated decrease in NLRP3 inflammasome activation (Hu et al., 2012). Likewise, EGCG can inhibit caspase-1 activation, and IL-1β and IL-18 secretion by blocking NLRP3 inflammasome activation in macrophages (Zhang et al., 2021). In NOD mice, EGCG increased plasma levels of the anti-inflammatory cytokine IL-10 (Fu et al., 2011), and resveratrol treatment altered the gene expression and trafficking of immune cells (Lee et al., 2011). In human primary adipocytes, resveratrol attenuated inflammation induced by conjugated linoleic acid, as evidenced by decreased extracellular signal-regulated kinase (ERK)1/2 activation, expression of cytokines (IL-6, IL-8, and IL-1β) and other inflammation-related proteins, ROS and intracellular Ca^2+^ (Kennedy et al., 2009).

In human trials, resveratrol supplementation reduced serum ALP and IL-6, and downregulated the expression of several pro-inflammatory genes in peripheral blood mononuclear cells in patients with T2DM (Tome-Carneiro et al., 2013). In another study, resveratrol treatment in obese subjects led to lower plasma leukocyte, TNF-α and IL-6 levels (although the latter was borderline) (Timmers et al., 2011). Skeletal muscle expression of genes involved in inflammation and cytokine signaling were also downregulated. Both resveratrol and EGCG lowered C-reactive protein levels in clinical studies (Bo et al., 2016; Hadi et al., 2020).

Some polyphenols mediate their anti-inflammatory effect by modulating the NFκB pathway. EGCG prevented TNF-α-induced expression of IL-8 by inhibiting IκB kinase (IKK) activity, degradation of Iκ-Bα, and thus NFκB activation (Chen et al., 2002). In HFD-rats treated with STZ, kaempferol treatment reduced protein expression of the NFκB p65 subunit, decreased phosphorylation of IKKα and IKKβ, and attenuated serum levels of TNF-α and IL-6 (Luo et al., 2015). Resveratrol prevented activation of NFκB by inflammatory agents and inhibited downstream gene expression and apoptosis by blocking phosphorylation of NFκB p65 and translocation of NFκB into the nucleus (Manna et al., 2000). Quercetin also blocked cholesterol-induced translocation of NFκB and decreased levels of IL-1β, TNF-α, IFN-γ, and granulocyte-macrophage colony-stimulating factor levels (Carrasco-Pozo et al., 2016). In STZ-rats, rutin decreased the levels of NFκB, IKKα, phosphorylated IKKα, IL-6 and TNF-α (Tian et al., 2016).

Inflammation can cause insulin resistance through Ser phosphorylation of insulin receptor substrate (IRS)-1, which inhibits insulin signaling (Mayer and Belsham, 2010). Quercetin can reverse palmitate-induced inflammation and insulin resistance by inhibiting ROS production, disruption of mitochondrial membrane potential, activation of NFκB, IL-6 and TNF-α production, and Ser phosphorylation of IRS-1, thus restoring insulin-induced Tyr phosphorylation of IRS-1 and insulin signaling (Guo et al., 2013). Quercetin, as well as rutin, also reduced Ser phosphorylation and increased Tyr phosphorylation of IRS-1 in the kidneys of fructose-fed rats (Hu et al., 2012). Phosphorylation of Akt and ERK1/2 were also increased. Likewise, treatment with kaempferol increased IRS-1 protein levels and decreased Ser phosphorylation in the liver of diabetic rats (Luo et al., 2015).

### ER Stress

Some polyphenols have also demonstrated an ability to protect against cytotoxicity by modulating ER stress. Dietary supplement of EGCG preserved islet structure in *db/db* mice, which was linked to reduced expression of several genes associated with ER stress (Ortsater et al., 2012). ECGC also protected MIN6 cells against palmitate-induced cytotoxicity by reducing the induction of ER stress-related gene expression (Ortsater et al., 2012).

In HFD-fed mice, both pomegranate extract and green tea extract prevented HFD-induced elevation of binding immunoglobulin protein (BiP), the spliced and unspliced forms of X-box binding protein 1, and activation of transcription factor 4, markers of the unfolded protein response, in skeletal muscle (Rodriguez et al., 2015).

In diabetic rats induced by STZ and a high-fat, high-carbohydrate diet, 16 weeks of treatment with grape seed proanthocyanidin extract decreased the expression of ER stress marker C/EBP homologous protein (CHOP), and the activities of JNK and caspase-12 in skeletal muscle (Ding, Dai, et al., 2013). In the pancreas, the extract reduced dilatation of the ER, the activity of JNK and protein levels of BiP, CHOP and caspase-12 (Ding, Zhang, et al., 2013). This was associated with decreased β-cell death and amelioration of pancreatic damage and dysfunction.

### Protective Effect in Mitochondria

Some polyphenols have demonstrated a protective effect in mitochondria, in turn preventing cellular dysfunction and apoptosis. Much of the research on this mechanism has been performed with resveratrol.

Mammalian target of rapamycin complex 1 (MTORC1), an inhibitor of autophagy, is excessively activated in INS1E β-cells that overexpress hA (Hernandez et al., 2018). These cells have an increased number of fissioned mitochondria and defective mitophagy. Resveratrol blocked MTORC1 activation by inhibiting nitration of TSC2, a marker of oxidative stress that activates MTORC1.

AMP-activated protein kinase (AMPK), sirtuin 1 (SIRT1) and peroxisome proliferator-activated receptor gamma coactivator 1α (PGC-1α) regulate mitochondrial biogenesis. Resveratrol supplementation in subjects with T2DM led to an increase in SIRT1 protein expression in skeletal muscle, as well as an increase in resting metabolic rate (Goh et al., 2014). Another study found that resveratrol increased AMPK phosphorylation, and SIRT1 and PGC-1α protein levels, and citrate synthase activity in skeletal muscle, suggesting improved mitochondrial function (Timmers et al., 2011). However, resveratrol is not a direct activator of SIRT1, and increased expression of SIRT1 is likely due to indirect effects (Pacholec et al., 2010). A study in AMPK-deficient mice revealed that resveratrol’s beneficial effects on glucose tolerance, insulin sensitivity, ROS reduction and mitochondrial biogenesis is dependent on AMPK (Um et al., 2010). Resveratrol-stimulated transcription of PGC-1α was dependent on AMPK but not SIRT1 (Um et al., 2010).

Quercetin also increased pancreatic gene expression of SIRT-1 and PGC-1α in the pancreas (Carrasco-Pozo et al., 2016). It also increased basal, maximal, and ATP-linked oxygen consumption rates, and increased the reserve capacity of mitochondria. Quercetin treatment protected MIN-6 cells from cholesterol-induced decreases in ATP levels, oxygen consumption and mitochondrial membrane potential, and inhibited cytochrome c release (Carrasco-Pozo et al., 2016). In *db*/*db* mice, curcumin supplementation increased hepatic AMPK expression but did not alter SIRT1 or PGC-1α levels, although protein activity was not measured (Jimenez-Flores et al., 2014). Genistein and EGCG have also been shown to activate AMPK (Hwang et al., 2005).

### Actions on the Insulin Signaling Pathway and Insulin-Like Effects

As described above, some polyphenols have been shown to normalize inflammation-induced perturbations in the insulin signaling pathway. Beyond this, polyphenols may have other effects, such as increasing insulin secretion, signaling and insulin-like effects on glucose uptake and production.

Several polyphenols are reported to increase glucose-stimulated insulin secretion (GSIS), including epicatechin (Hii and Howell, 1984), quercetin (Hii and Howell, 1985; Youl et al., 2010; Lee et al., 2021), genistein (Liu et al., 2006), oleuropein (Wu et al., 2017), and curcumin (Best et al., 2007). However, their reported mechanism of action varies. Epicatechin and quercetin may augment GSIS by suppressing Ca^2+^ efflux and increasing Ca^2+^ uptake (Hii and Howell, 1985; Youl et al., 2010), or by inhibiting cAMP phosphodiesterase (PDE) (Beretz et al., 1978). The effect of genistein was mediated by intracellular accumulation of cAMP due to adenylyl cyclase activity, rather than inhibition of PDE (Liu et al., 2006). Oleuropein stimulated GSIS by activation of the ERK/MAPK pathway (Wu et al., 2017), and curcumin by activation of the volume-regulated anion channel and Cl^−^ efflux (Best et al., 2007). Not all polyphenols stimulate insulin release. In fact, chrysin and naringenin can inhibit insulin secretion (Hii and Howell, 1985), while there is conflicting evidence as to whether resveratrol is an insulin secretagogue or inhibits secretion (Chen et al., 2007; Szkudelski and Szkudelska, 2011).

Some polyphenols are reported to upregulate components of the insulin signaling pathway. Pancreatic expression of Ins-1, ngn-3 and PDX1, were increased by CPP extract in rats with alloxan-induced diabetes, while IRS-1 expression was increased in the liver (Iftikhar et al., 2020). Resveratrol supplementation in T2DM patients increased the ratio of phosphorylated Akt to unphosphorylated Akt in platelets (Brasnyo et al., 2011). Curcumin decreased plasma levels of GSK-3β, overexpression of which is associated with insulin resistance, in subjects at risk of developing T2DM (Thota et al., 2020).

Polyphenols may be able to increase glucose uptake in various tissues. Green tea supplementation increased the number of insulin binding sites (Wu, Juan, Ho, et al., 2004) and glucose uptake (Wu, Juan, Ho, et al., 2004) in rat adipocytes, and glucose transporter (GLUT)-4 protein levels in the adipocytes of mice fed a high fructose diet (Wu, Juan, Hwang, et al., 2004). Quercetin increased GLUT-4 expression in the adipose tissue, skeletal muscle, and serum of mice with alloxan-induced diabetes (Alam et al., 2014). Resveratrol increased phosphorylated Akt and GLUT-4 protein expression in the skeletal muscle of STZ-treated rats (Chi et al., 2007). In C_2_C_12_ myotube cells, resveratrol increased glucose uptake, which was blocked by PI3K inhibition, suggesting resveratrol increases GLUT-4 expression and glucose uptake by skeletal muscle via a PI3K-Akt pathway-dependent mechanism (Chi et al., 2007).

The ability of polyphenols to upregulate GLUT-4 may also be via activation of AMPK. Resveratrol (Penumathsa et al., 2008; Do et al., 2012; Goh et al., 2014) and curcumin (Na et al., 2011) have been shown to activate AMPK with a concomitant increase in GLUT-4 levels or translocation to the cell membrane. Curcumin also inhibited pyruvate dehydrogenase kinase 4 expression and phosphorylation of glycogen synthase in skeletal muscle, suggesting increased glucose oxidation and glycogen synthesis (Na et al., 2011).

Evidence suggests that several polyphenols can inhibit hepatic glucose production by reducing gluconeogenic enzyme activity and upregulating glycolytic enzyme activity. EGCG inhibits hepatic glucose production (Waltner-Law et al., 2002) and expression of gluconeogenic enzymes phosphoenolpyruvate carboxykinase (PEPCK) (Waltner-Law et al., 2002; Koyama et al., 2004; Wolfram et al., 2006), glucose 6-phosphatase (G6Pase) (Waltner-Law et al., 2002; Koyama et al., 2004; Wolfram et al., 2006) and fructose-1,6-bisphosphatase 1 (FBPase) (Wolfram et al., 2006) in H4IIE cells. Conversely, it upregulated expression of the glycolytic enzyme phosphofructokinase and GLUT-1 (Wolfram et al., 2006). Similarly, resveratrol decreased hepatic G6Pase and PEPCK activity, and increased glucokinase activity and pyruvate kinase expression (Do et al., 2012). Curcumin supplementation in *db*/*db* mice elevated hepatic glucokinase activity and lowered G6Pase and PEPCK activity (Seo et al., 2008). Quercetin reduced G6Pase and FBPase activity in liver, kidney, and skeletal muscle (Alam et al., 2014). Quercetin also increased hepatic hexokinase activity in alloxan-induced diabetic mice (Alam et al., 2014) and STZ rats (Vessal et al., 2003). Likewise, CPP extract decreased G6Pase and FBPase activity, and restored the activities of G6P dehydrogenase and hexokinase in alloxan-treated rats (Iftikhar et al., 2020). In STZ-rats, kaempferol demonstrated the ability to reduce hepatic glucose production and pyruvate carboxylase activity, and increase glucokinase activity and glycogen storage (Alkhalidy et al., 2018).

Taken together, available evidence suggests that some polyphenols may augment GSIS, upregulate the insulin signaling pathway, increase glucose uptake and utilization, and reduce endogenous glucose production, which may partly explain their reported blood glucose-lowering and glucose tolerance-enhancing effects. Some of these effects may also be explained by improved β-cell survival and function rather than a direct metabolic effect.

### Lipid Levels and Metabolism

High circulating levels of FFA are known to increase insulin resistance. FFA are metabolized through β-oxidation to produce acetyl-CoA and ATP, or to make other metabolites such as diacylglycerides and ceramides, which are suggested to decrease insulin sensitivity (Corcoran et al., 2007). Supplementation with several polyphenols has been shown to lower circulating levels of FFA, triglycerides and/or cholesterol in diabetic animal models or human subjects, including EGCG (Wolfram et al., 2006; Roghani and Baluchnejadmojarad, 2010), resveratrol (Timmers et al., 2011; Do et al., 2012), quercetin (Vessal et al., 2003; Carrasco-Pozo et al., 2016; Eitah et al., 2019), kaempferol (Luo et al., 2015), curcumin (Seo et al., 2008; Na et al., 2011), and oleuropein (Lockyer et al., 2017; Araki et al., 2019).

This lipid-lowering effect of polyphenols may be due to alteration of the expression and activity of enzymes involved in lipid uptake and metabolism. EGCG downregulated several genes involved in lipid metabolism in rat H4IIE hepatoma cells, and in *db/db* mice it increased expression of acyl-CoA oxidase 1 and carnitine palmitoyl transferase (CPT)-1β in liver and adipose tissue (Wolfram et al., 2006).

Curcuminoid treatment in patients with T2DM increased serum lipoprotein lipase (LPL) activity (Na et al., 2013). Since LPL converts triglycerides into FFA, it was suggested that curcuminoids increase uptake and utilization of FFA in tissues. In L6 myotubes, curcumin increased CD36 and CPT-1 expression, and phosphorylation of acetyl-CoA carboxylase, suggesting that curcumin lowers FFA levels by increasing uptake and β-oxidation in skeletal muscle (Na et al., 2011). This effect was mediated by activation of AMPK. Likewise, in *db*/*db* mice, curcumin elevated LPL activity in skeletal muscle (Seo et al., 2008). This was also associated with a reduction of the elevated levels of hepatic β-oxidation and lipid-regulating enzyme activity in *db*/*db* mice, including fatty acid synthase, CPT, HMG-CoA reductase, and acyl-CoA: cholesterol acyltransferase.

### Summary

The pleiotropic effects of many polyphenols may be useful in combatting T2DM, especially considering its equivocal etiology, multiple dysfunctional cellular pathways, and uncertainty over which are causes and which are consequences of the disease. Polyphenol treatment could have pancreatic and extra-pancreatic effects. Inhibition of amyloid formation, antioxidant, anti-inflammatory, mitochondrial and ER-stress protective effects could preserve β-cells, while anti-inflammatory, serum lipid lowering, and insulin-like effects (particularly on liver enzyme activity) could improve whole-body insulin sensitivity and help ameliorate hyperglycemia.

## Areas for Further Research

### Bioavailability and Metabolism

The bioavailability and metabolism of polyphenols should be considered in future studies. Many available studies have shown an effect after oral administration in animal models, but clinical studies have provided variable results, which could be due to dose, methodology or differences in bioavailability or metabolism.

Some polyphenols are bioavailable in their native form. ECGC is found in plasma in free form following oral administration, albeit at low concentrations (Andreu-Fernandez et al., 2020). Resveratrol can be absorbed in its native state but can also undergo considerable microbial metabolism before absorption (Bode et al., 2013). However, some polyphenols are largely metabolized before absorption. Rutin is metabolized by microbiota before the metabolites are absorbed in both humans and rodents (Baba et al., 1981; Baba et al., 1983).

There is significant interspecies variation in the metabolism of xenobiotics. For example, resveratrol and dihydroresveratrol in human subjects after oral administration have been observed at higher plasma levels than those obtained in mice given considerably higher doses (Timmers et al., 2011). Furthermore, interindividual differences in the metabolism and absorption of compounds may underlie clinical variability. The level of bioavailability of resveratrol between individuals is inconsistent. Microbial metabolism between individuals can vary greatly, which may explain the conflicting results from human trials (Bode et al., 2013; Poulsen et al., 2013). Substantial variability in absorption and stability of polyphenols can also occur due to the fed/fasted state of subjects and whether it is ingested with other nutrients (Andreu-Fernandez et al., 2020).

The bioactivity of most metabolites of polyphenolic compounds is poorly understood. The plasma level of free, unconjugated quercetin following rutin supplementation is reported to be low (Erlund et al., 2000). Despite this, an antidiabetic effect has been reported following oral rutin administration *in vivo*, raising the question of whether one or more metabolites are responsible (Aitken et al., 2017).

So far, there has been little published research on the activities of metabolites and their link to the clinical efficacy of the parent compound. Bioavailability and metabolism should be considered in the design of future intervention studies (Manach et al., 2005). An avenue for further research may be to design drugs based on polyphenolic structures that may be more stable or targeted in the body, or with novel delivery systems, to improve absorption and efficacy.

### Inhibition of Amylin Aggregation at Lipid Membranes

The inhibitory effects of polyphenols on hA aggregation are often studied *in vitro* in aqueous solutions; however, this does not accurately recapitulate the lipid membrane environment in which hA aggregates physiologically. Studies utilizing a variety of membrane-mimetic model systems have demonstrated that lipid membranes containing an anionic charge accelerate fibril formation (Knight et al., 2006; Sasahara et al., 2010; Christensen and Schiott, 2019). However, few studies have reported on the ability of polyphenols to inhibit hA aggregation in such an environment, with results only for EGCG and resveratrol reported to our knowledge. EGCG had greatly reduced efficacy (∼50%) in inhibiting fibril formation and lost its ability to disaggregate pre-formed fibrils in the presence of a phospholipid membrane (Engel et al., 2012). By contrast, resveratrol inhibited hA aggregation even in the presence of lipids (Evers et al., 2009; Mishra et al., 2009). Further investigation into whether other polyphenols retain their inhibitory effect on amyloid formation in a lipid membrane environment would be valuable.

### Potential as a Multi-Target Therapeutic

The pathogenesis of T2DM may overlap with that in other diseases, in particular Alzheimer’s disease which has been suggested to be a third type of diabetes (Steen et al., 2005). This opens the possibility that polyphenols, especially with their pleiotropic effects, may be an effective treatment for several diseases. Many polyphenols act as general amyloid inhibitors and are effective against amyloid-β (Alzheimer’s disease), α-synuclein (Parkinson’s disease) and prion proteins (Porat et al., 2006).

The antioxidant and anti-inflammatory activities of polyphenols have non-disease specific, beneficial effects that may be especially useful for those diseases with a complicated and varied etiology like T2DM. Research into the use of polyphenols in the treatment or prevention of cancer, Alzheimer’s disease, Parkinson’s disease, cardiovascular disease, hypertension, and other conditions is ongoing (Khursheed et al., 2020). Additionally, with their well-tolerated safety profile there is the possibility for use of polyphenolic compounds as a preventative as well as therapeutic treatment.

### Methodology

Future studies should carefully consider the methodologies they use. As discussed, ThT fluorescence should not be used as the sole method to measure inhibition of hA aggregation by compounds due to the possibility of competitive binding (Daval et al., 2010). Circular dichroism, atomic force microscopy or other methods should be utilized to verify results. High resolution nuclear magnetic resonance or mass spectrometry techniques could be used to probe the specific interaction of compounds with hA, which could provide vital insights into the binding mechanism and facilitate rational drug design based on polyphenolic compounds. For *in vivo* studies, the appropriateness of the animal model used must also be considered, as many models recapitulate part of but not the entirety of T2DM. Many commonly used models, such as STZ or alloxan-induced diabetic animals, are more reminiscent of T1DM than T2DM. Few studies utilize transgenic mice that express the human variant of amylin which forms amyloid (Aitken et al., 2017), and are thereby unable to measure the effectiveness of the compound against amyloid formation that evidence suggests is involved in T2DM pathogenesis. Similarly, the use of cell lines that overexpress hA in *in vitro* studies, rather than applying exogenous hA to cells, may be more physiologically relevant.

## Conclusion and Perspectives

For decades, T2DM was considered as predominantly a disease caused by insulin resistance and therapies focused on lowering blood glucose levels. However, in recent years there has been a paradigm shift, from a glucose-centric to a β-cell-centric view of T2DM (Saisho, 2020). This evolution recognizes that T2DM occurs only when β-cell function has failed. The etiology underlying this process is unclear; however, evidence suggests that cytotoxic hA oligomers, oxidative stress, inflammation, ER stress and mitochondrial dysfunction all play their part, even if a single causative mechanism (if there is indeed one) is yet to be determined. Therefore, agents that target multiple pathways may lead to better therapeutic outcomes.

In this regard, polyphenols are promising candidates with their ability to inhibit amyloid formation (Porat et al., 2006). As hA oligomers have been shown to contribute to oxidative stress (Zraika et al., 2009), inflammation (Masters et al., 2010; Zhang et al., 2011), ER stress (Casas et al., 2007; Casas et al., 2008) and mitochondrial dysfunction (Li et al., 2011) in addition to being directly cytotoxic themselves (Kayed et al., 2003; Zhang et al., 2008), they are a major therapeutic target with a multi-pronged effect. Additionally, polyphenols have more direct effects on reducing oxidative stress and inflammation, as well as modulating other cellular pathways with beneficial metabolic effects. Thus, polyphenols may have beneficial effects on both β-cell survival and whole-body insulin sensitivity.

Both scientists and the public have shown strong interest in polyphenols, their potential health benefits, and their use as treatments for a multitude of diseases, especially surrounding EGCG, resveratrol, and curcumin. However, hopes for a new effective therapeutic treatment based on the findings summarized here have yet to come to fruition. Clinical trials have shown some encouraging but controversial results. The greatest challenge appears to be achieving a consistent therapeutic effect.

The current impasse may be due to incomplete understanding of the molecular basis of polyphenol action, alongside the complexity of multifactorial diseases such as T2DM. Further research could usefully focus on the following: 1) the bioavailability of compounds in humans and establishing a therapeutic dose range; 2) the identity and activity of metabolites; 3) consideration of methodology for *in vitro* studies and *in vivo* trials and ensuring that clinical trials examine appropriate parameters; 4) design of compounds or delivery systems with greater stability and improved and reproducible efficacy.

Natural polyphenols remain an active area of research for many diseases (Khursheed et al., 2020). Improved compounds and robust study designs will enable greater understanding of how to utilize these interesting, multifunctional compounds.

## Glossary

AIF: apoptosis-inducing factor
AMPK: AMP-activated protein kinase
BiP: binding immunoglobulin protein
CHOP: C/EBP homologous protein
CPP: caesalpinia bonduc
CPT: carnitine palmitoyl transferase
EGCG: epigallocatechin gallate
ER: endoplasmic reticulum
ERK: extracellular signal-regulated kinase
FBPase: fructose-1,6-bisphosphatase
FFA: free fatty acids
G6Pase: glucose 6-phosphatase
GLUT: glucose transporter
GPx: glutathione peroxidase
GSIS: glucose-stimulated insulin secretion
GSK-3β: glycogen synthase kinase-3β
GST: glutathione S-transferase
hA: human amylin
HbA1c: hemoglobin A1c
HFD: high fat diet
HOMA-IR: homeostatic model assessment of insulin resistance
IFN-γ: interferon γ
IKK: IκB kinase
IL: interleukin
iNOS: inducible nitric oxide synthase
IRS-1: insulin receptor substrate
JAK2: janus kinase 2
JNK: c-Jun N-terminal kinase
LKB1: liver kinase B1
LPL: lipoprotein lipase
MAPK: mitogen-activated protein kinase
MTORC1: mammalian target of rapamycin complex 1
NA: nicotinamide
NFκB: nuclear factor κ B
NLRP3: nod-like receptor protein 3
NOD: non-obese diabetic
NOX: NADPH oxidase
PDE: cAMP phosphodiesterase
PDX1: pancreatic and duodenal homeobox 1
PEPCK: phosphoenolpyruvate carboxykinase
PGC-1α: peroxisome proliferator-activated receptor gamma coactivator 1α
PI3K: phosphoinositide 3-kinase
PKA: protein kinase A
RAGE: receptor for advanced glycation end-products
ROS: reactive oxygen species
SIRT1: sirtuin 1
SOD: superoxide dismutase
STAT3: signal transducer and activator of transcription 3
STZ: streptozotocin
T2DM: type 2 diabetes mellitus
ThT: thioflavin T
TNF-α: tumor necrosis factor α
TRPV4: transient receptor potential cation channel subfamily V member 4
